# Update on non-infectious uveitis treatment: anti-TNF-alpha and beyond

**DOI:** 10.3389/fopht.2024.1412930

**Published:** 2024-08-02

**Authors:** Khushboo Chauhan, Mudit Tyagi

**Affiliations:** ^1^ Saroja A Rao Centre for Uveitis, L V Prasad Eye Institute, Hyderabad, India; ^2^ Smt. Kanuri Santhamma Centre for Vitreo-Retinal Diseases, L V Prasad Eye Institute, Hyderabad, India

**Keywords:** non-infectious uveitis (NIU), TNF alpha inhibitors, mTOR inhibitors, rituximab, JAK inhibitors, PDE-4 inhibitors, interleukin inhibitors, ACTH analogues

## Abstract

Non-infectious uveitis (NIU) encompasses a range of conditions marked by inflammation within various layers of the eye. NIU is a significant contributor to irreversible vision loss among the working-age population in developed countries. The aim of treating uveitis is to manage inflammation, prevent its recurrences and to restore or salvage vision. Presently, the standard treatment protocol for NIU involves initiating corticosteroids as the primary therapeutic agents, although more aggressive approaches and steroid sparing agent may be necessary in certain cases. These advanced treatments option include synthetic immunosuppressants like antimetabolites, calcineurin inhibitors and alkylating agents. For patients who exhibit an intolerance or resistance to corticosteroids and conventional immunosuppressive therapies, biologic agents have emerged as a promising alternative. Notably, among the biologic treatments evaluated, TNF-α inhibitors, anti-CD20 therapy and alkylating agents have shown considerable efficacy. In this review, we delve into the latest evidence surrounding the effectiveness of biologic therapy and introduce novel therapeutic strategies targeting immune components as potential avenues for advancing treatment of NIU.

## Highlights

- The objective of this manuscript is to provide a comprehensive review of the current understanding and advancements in the treatment of non-infectious uveitis, with a specific focus on the role of anti-TNF alpha therapies and emerging treatment options. The manuscript aims to highlight the efficacy, safety, and mechanisms of action of anti-TNF alpha agents, and explore alternative and adjunctive therapies that have shown promise in recent research. However, the manuscript focuses exclusively on agents currently being tested in human clinical trials. Therapies still in the experimental stage with animal subjects are not included in this study.
- The key areas of focus include an overview of anti-TNF alpha agents (e.g., infliximab, adalimumab, etanercept), novel biologics such as interleukin inhibitors, JAK inhibitors, mTOR inhibitors, interferons, and various other drugs. The manuscript explains their mechanisms of action in the context of uveitis, supported by pictorial representations. It also includes information on clinical trials, dosages, and adverse events for each respective agent.
- The primary audience for this manuscript includes ophthalmologists, immunologists, rheumatologists, clinical researchers, and healthcare professionals involved in the management of uveitis. Additionally, it will be valuable to pharmaceutical scientists and policymakers interested in the development and regulation of new therapeutic agents for ocular inflammatory diseases.

## Introduction

The term “uvea” finds its roots in the Latin word “uva,” alluding to the visual similarity of the uveal tissue beneath the sclera to a “black grape” (1). Uveitis encompasses a wide range of inflammatory conditions affecting the intraocular structures including the iris, ciliary body, and choroid. Additionally, it may involve adjacent eye components, including the cornea, vitreous humor, retina, and optic nerve. Uveitis is a prevalent global cause of blindness, with its onset attributed to either infectious or non-infectious factors (2). In developing countries, infectious uveitis is more prevalent, constituting 30–50% of all cases, while in Western countries, most cases are attributed to autoimmune causes. The predominant causes of Non-Infectious Uveitis (NIU) include HLA-B27 associated anterior uveitis, Vogt–Koyanagi–Harada syndrome, sarcoidosis, sympathetic ophthalmia, serpiginous choroiditis, birdshot chorioretinopathy (BSCR), Behçet’s disease (BD) and multifocal choroiditis (3).

The management of uveitis depends upon the extent of inflammation, the existence of risk factors, and the presence of complications. Initiation of treatment should occur promptly upon diagnosis, often following a step-by-step approach. This approach begins with the least aggressive treatments and progressively escalates to more intensive measures, aiming to achieve remission of inflammation. Presently, the primary approach to treat uveitis involves the use of corticosteroids, aimed at reducing the severity of inflammation (4, 5). While corticosteroids are frequently effective, their prolonged use is constrained by potential ocular and systemic side effects (6, 7). Other treatment options for both primary and secondary Non-Infectious Uveitis (NIU) encompass traditional immunosuppressants such as cyclosporine, methotrexate, azathioprine, sulfasalazine, and mycophenolate mofetil. Nonetheless, a significant proportion of uveitis cases cannot be adequately managed solely with corticosteroids and immunosuppressants (8). The introduction of different biologics and other innovative emerging treatment modalities have now added to the armamentarium of treatment modalities for the treatment of NIU. This review aims to explore the use of biologic agents like TNF alpha inhibitors and other agents and intravitreal drug delivery in the treatment of non-infectious uveitis.

## Method of literature search

A comprehensive literature review on PubMed, ePub,Cochrane library databases and Google Scholar utilizing the keywords “non-infectious uveitis,” “biologicals,” and “TNF-alpha inhibitors.” was done. A total of 410 articles were found related to the management of non-infectious uveitis. Literature search was not limited by the year of publication and all the pertinent original articles, reviews, case reports, case series, and hypotheses published until February 2024 were systematically examined and included. Additionally, reference lists of relevant articles when clinically relevant and pertinent to the scope of this review were also reviewed. This review exclusively included 146 articles that were published in the English language. Furthermore, the review also encompasses drugs that have undergone human trials or have been approved for human use. However, treatment modalities still undergoing animal experiments were excluded from this review.

## Anti-TNF alpha inhibitors

**Figure 1:**
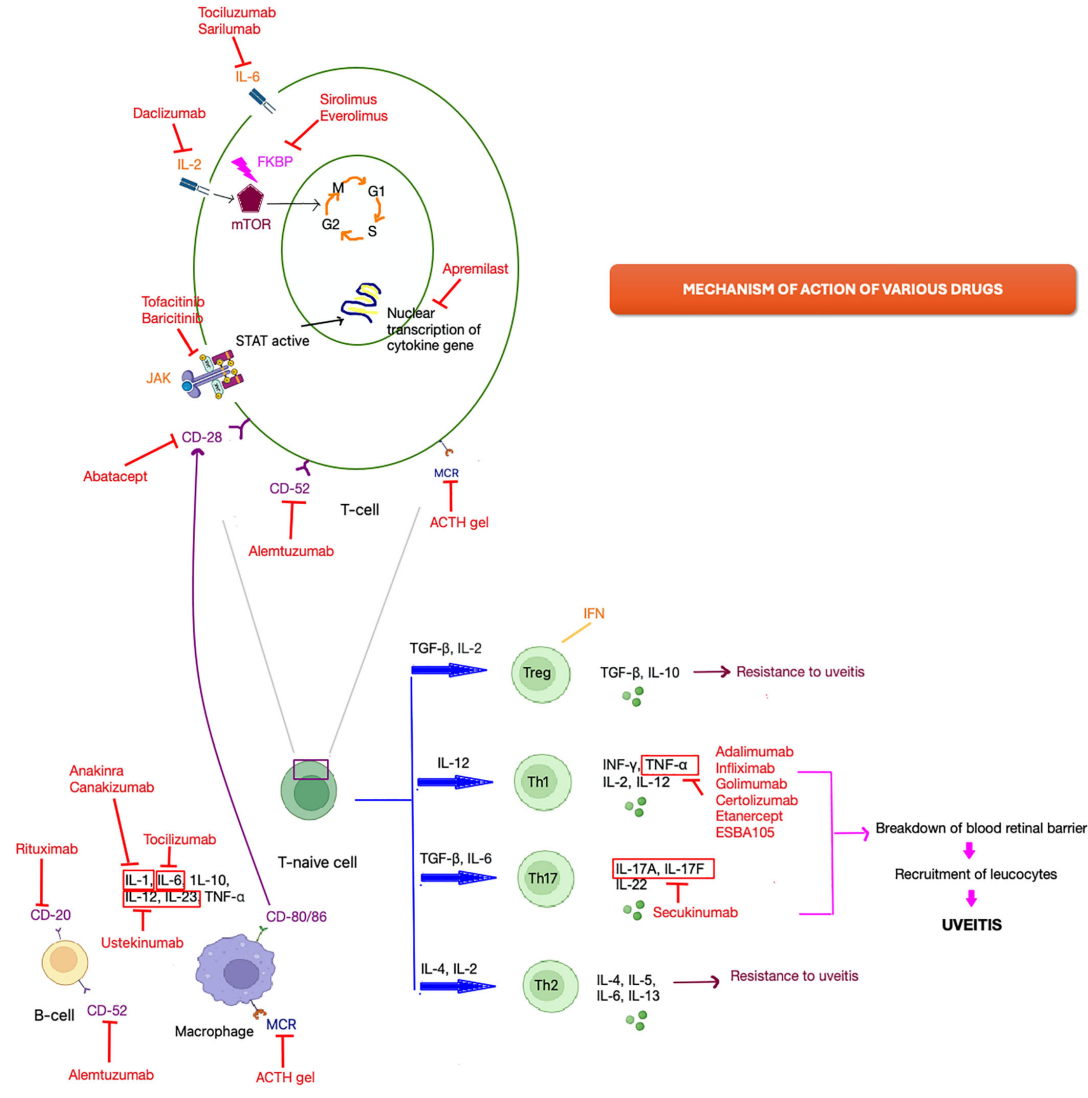
Schematic representation of the mechanism of action of various drugs in NIU.

Tumor necrosis factors constitute a cluster of cytokines generated by CD4+ lymphocytes, activate macrophages and natural killer cells. These cytokines play a crucial role in triggering inflammation and apoptosis, as well as impeding viral replication (9, 10). The pro-inflammatory cytokine TNF-α is believed to be a pivotal factor in uveitis inflammation, with upregulated levels in both aqueous humor and serum among uveitis patients. Tumor necrosis factor α attaches to distinct membrane receptors, namely TNF-α receptors I and II which are situated on the surface of pigment epithelium cells in the iris, ciliary body, and retina (10). Additionally, TNF-α has been identified to elevate the production of vascular endothelial growth factor (VEGF) in choroidal endothelial cells, with VEGF being accountable for macular edema in individuals with uveitis (8, 11). This accounts for the positive result observed in TNF- α inhibitors therapy, which effectively lowers the inflammation and plasma VEGF levels by inhibiting TNF-α production in the management of uveitic macular edema (10, 12). The mechanism of various drugs is illustrated in **Figure 1**. Anti-TNF-α agents are recommended in both adult and pediatric population (13).

Currently, there are five biologic agents targeting TNFα that are approved for treating various conditions such as rheumatoid arthritis, juvenile idiopathic arthritis (JIA), psoriatic arthritis, inflammatory bowel disease and ankylosing spondylitis. These medications include adalimumab (Humira^®^), infliximab (Remicade^®^), golimumab (Simponi^®^), certolizumab (Cimzia^®^), and etanercept (Enbrel^®^) (14). Apart from their approved indications, anti-TNFα agents are also utilized off-label in conditions like non-infectious uveitis, sarcoidosis, Behçet disease, adult-onset Still’s disease, pyoderma gangrenosum, as well as in patients with TNF receptor-associated periodic fever syndrome (TRAPS) (15, 16). Adalimumab is the only TNF-α inhibitor which has gained approval for management of non-infectious uveitis.

Adalimumab, a fully human anti-TNF-α monoclonal antibody, has gained approval for the management of various immune-mediated inflammatory conditions, including non-infectious intermediate, posterior, and panuveitis (10). The drug was approved for use in adult and pediatric population in 2016 and 2017 respectively (17). The dosage and other adverse effects of various TNF-α inhibitors are summarized in **Table 1**. The HOT study showed that tapering of adalimumab should be approached with caution, as faster tapering has been linked to higher recurrence rates. It has been observed that with every 10 weeks of inactivity preceding the attempt to taper, there was a 3.6% reduction in the recurrence rate. “Pediatric and younger patients are more prone to recurrence. Additionally, patients of white and Arab descent tend to have a higher likelihood of recurrence compared to other populations (24). Various studies which have proven the efficacy of adalimumab are enlisted in **Table 2**. A biosimilar of adalimumab called SB5 has recently gained approval for treating non-infectious uveitis (NIU) and various other autoimmune conditions including rheumatoid arthritis (RA), juvenile idiopathic arthritis (JIA), and inflammatory bowel disease (IBD), among others (36).

**Table 1:** Pharmacotherapy used in management of non-infectious uveitis.

		Description	Dose	Adverse effect
*TNF-α inhibitors*	Adalimumab (Humira)	Humanized antibody	Adult Loading dose:80mg Maintenance dose:40 mg every 2 weeks Pediatric (<16 years) minimum of 24 mg/m2, with a weekly maximum of 40 mg (18, 19).	Autoimmune diseases, infections, injection site reaction Arthralgia, nasopharyngitis, headache
	Infliximab (Remicade)	Chimeric antibody	Adult 3–5 mg/kg every 6–8 weeks depending on the severity Pediatric (<16 years) Loading dose: 3 and 5 mg/kg at weeks 0, 2, and 6 Maintenance dose: 7.5 mg/kg/dose every 4 to 8 weeks; maximum dose - 20 mg/kg (19, 20)	Autoimmune diseases, infections, injection site reaction Lupus like reaction
	Certolizumab (Cimzia)	Recombinant humanized antibody	Loading dose: 400mg at 0,2,and 4 weeks Maintenance dose: 200 mg per week (21)	Autoimmune diseases, infections, injection site reaction Lupus like reaction, anaphylactic Rx, generalized skin Rx
	Etanercept (Enbrel)	Dimeric protein (Fc portion of human IgG & a portion of the ligand-binding site of the TNF receptor p75)	Adult 50 mg once per week or 25 mg twice a week sc. Pediatric >2 years old, 0.4 mg/kg (max 25 mg/dose) twice a week Or 0.8 mg/kg (max 50 mg) once weekly (22)	Autoimmune diseases, infections, injection site reaction IBD flares, uveitis, granulomatous disease
	Golimumab (Simponi)	Humanized antibody	50 mg every 4 weeks sc (20, 23)	Autoimmune diseases, infections, injection site reaction Anemia, fever and nausea
IL inhibitors	Tocilizumab Anakinra Canakizumab Secukinumab Ustekinumab	IL-6 inhibitors IL-1 inhibitor (recombinant form) IL-1 inhibitor (Humanized monoclonal antibody) IL-17 inhibitor (Human monoclonal antibody) IL-12 inhibitor (Human monoclonal antibody)	Induction- monthly IV regimen of 4 mg/kg, with the option to escalate to 8 mg/kg monthly depending on the therapeutic response 100 mg/d sc 50 mg SC every 8 wk to 300 mg every 4 wk 30 mg/kg iv every 4 weeks 45 mg SC every 4 week, subsequently every 8 week, then every 12 week	GI symptoms, infections Bacterial and viral infections, neutropenia and skin reactions Bacterial and viral infections, neutropenia and skin reactions URTI and headache GI symptoms, infections, infusion reactions, headache, flu like symptoms
Anti-CD20 inhibitors	Rituximab	Chimeric (monoclonal antibody)	1000 mg/d IV on day 1 and 15, repeated after 6 months or 375 mg/m^2^ body surface/week for 8 week and then monthly for 4 months	Infusion reactions, myelosuppression, GI symptoms, infections, progressive multifocal leukoencephalopathy
Selective costimulation modulator	Abatacept	Recombinant fusion protein	125 mg SC or 10 mg/kg IV at wk 0, 2, and 4; then every 4 wk	GI symptoms, infections, night sweats, flu-like symptoms, urinary symptoms, skin rashes
Anti CD-25	IL-2 receptor alpha subunit blocker	Humanized monoclonal antibody	Induction: 2 mg/kg sc (max 200 mg) every 2 weeks for 1 month; Maintenance: 1/mg/kg (limit 100 mg) sc monthly	Herpes zoster, rash, liver enzymes derangement and palpitations
Anti-CD 52	Alemtuzumab	Humanized monoclonal antibody	30 mg intravenously, 3 days in a week for 12 weeks	allergic reactions, mild flu-like symptoms, and gastrointestinal irritation.
JAK inhibitors	Tofacitinib Baricitinib	JAK 1 and 3 inhibitors JAK 1 and JAK 2 inhibitors	10 mg/day oral 4 mg/day oral	Infections, GI symptoms, abnormal liver tests, hypertension
Interferons	IFN-α 2a IFN-α 2b IFN-β	Recombinant form Pegylated form Recombinant form Recombinant form	3–6 MIU SC or IM daily to 3 times per week 90 or 180 μg weekly 3–4.5 MIU per day SC for 2 wk, tapering to 3 times per week 44 μg SC 3 times per week	GI symptoms, infections, alopecia, myelosuppression, abnormal thyroid function, depression, abnormal liver function tests
mTOR inhibitors	Sirolimus		Intravitreal 440 μg	Under trial
PDE-4 inhibitors	Apremilast		30 mg BD for 12 weeks	Under trial
ACTH analogues	ACTH gel			Under trial
Immunoglobulin		Polyclonal human IgG	1.5–2.5 g/kg IV over 3 days, repeated accordingly	Fever, hypertension, eczema

**Table 2:** Clinical studies showing the efficacy of various pharmacotherapy in non-infectious uveitis.

Drug	Description	Study	Type of study	Participants	Results
Adalimumab	anti-TNF-α humanized antibody	VISUAL 1 (11) VISUAL 2 (25) VISUAL 3 (26) SYCAMORE (27) ADJUVITE (28)	Multicentered, randomized placebo controlled trial Multicentered, randomized, double-masked, placebo controlled trial Open-label, multicentric extension study of VISUAL 1 & VISUAL 2 multicenter, double-blind, randomized, placebo-controlled trial multicenter, double-blind, randomized, placebo-controlled trial	217 226 371 90 32	Efficacy of adalimumab as glucocorticoid sparing agent was proven in active non-infectious uveitis Efficacy of adalimumab was proven in corticosteroid dependent inactive non-infectious uveitis Safety and efficacy of adalimumab in patients with non-infectious(active and inactive) intermediate, posterior, or panuveitis. Efficacy of adalimumab was proven in treatment of JIA associated uveitis Efficacy of adalimumab in patients with early onset, chronic anterior uveitis, associated with JIA, in case of inadequate response to topical therapy and Methotrexate.
Tofacitinib and Baricitinib	JAK inhibitors	Miserochhi et al (29)	Case series	4	• Efficacy of JAK inhibitors is shown in JIA refractory to DMARDs,TNF-α inhibitors • Resolution of ocular inflammation and macular edema
Abatacept	Selective costimulation modulator	Zulian et al (30)	Case series	7	• Efficacy of Abatacept is shown in JIA refractory to TNF-α inhibitors • Reducing ocular inflammation & preventing relapse
Interferons Interferon α-2A Interferon α- 2b Interferon-β	Recombinant IFN Recombinant IFN Recombinant IFN	Bodaghi et al (31) Kotter et al (32) Deuter et al (33) Simoni et al (34) Celiker et al (31) Becker et al. (35)	Retrospective study open, non-randomized, uncontrolled prospective study Retrospective study Retrospective study Case series Retrospective observational case series	45 50 24 37 4 13	• Reducing ocular inflammation & preventing relapse in Behcet’s & other uveitis • Reduces inflammation & relapses & maintain remission in Behcet’s disease • Reduction in chronic refractory macular edema • Effective in treating post-uveitic refractory ME • Efficacy in Behcet’s uveitis • Efficacy in multiple sclerosis associated uveitis
Sirolimus	mTOR inhibitors	SAVE 1 SAVE 2 SAKURA	Prospective, randomized, phase 1 interventional trial Prospective, randomized, phase 2 interventional trial Prospective, randomized, multi-national study	30 24 592	• Intravitreal/subconjunctival route are tolerated by the patient(Intravitreal>subconjunctival) • Low dose and high dose of Sirolimus were found to decrease vitreous haze whereas low dose(monthly) >high dose(2 monthly) in reducing uveitic macular edema • Intravitreal 440 μg > 44 μg in reducing vitreous haze and preserving VA

Infliximab, certolizumab, golimumab, and etanercept are among several other TNF-α inhibitors that do not have FDA approval for the treatment of non-infectious uveitis. Nevertheless, their effectiveness is supported by substantial evidence. Due to being fully humanized antibodies, adalimumab and golimumab exhibit lower immunogenic potential compared to chimeric antibodies (37).

Infliximab, a chimeric monoclonal antibody, has demonstrated its efficacy in treating Behcet’s disease and refractory cases of non-infectious uveitis (NIU) (38–40). Golimumab has proven effective in patients with NIU resistant to adalimumab or infliximab, making it typically reserved for treating this subset of non-responders (23, 41, 42). Certolizumab and Golimumab are reserved for cases where other anti-TNF-α agents have proven ineffective (43).

The mechanism of action of etanercept (Enbrel^®^) differs from other anti-TNF-α agents. It is a dimeric protein comprising an Fc portion of human IgG and a ligand-binding site portion of the TNF receptor p75 (44). Etanercept is approved for treating juvenile idiopathic arthritis (JIA) in children over 2 years old (22).

The primary adverse effects associated with anti-TNF-α agents include the onset of autoimmune diseases, heightened susceptibility to infections, notably tuberculosis and histoplasmosis, among others. Injection site reactions have also been reported (10). Furthermore, anti-TNF-α blockers have been linked to an elevated risk of malignancies, particularly lymphomas (45), although subsequent studies have largely refuted this association (46). Additionally, they have been implicated in the onset or exacerbation of demyelinating disorders such as multiple sclerosis (47).

ESBA105 is a topically administered TNF-α inhibitor. It comprises a single-chain antibody fragment known for its potent inhibition of TNF- α. Unlike full monoclonal antibodies, these fragments lack constant domains, representing the smallest functional units of antibodies. Their small size offers potential advantages for topical administration, facilitating better penetration through the corneal surface (48, 49). Functioning through the same pathways as full anti-TNF antibodies like infliximab and adalimumab, ESBA105 has demonstrated comparable efficacy (50).

A considerable number of patients encounter a loss of response while undergoing maintenance treatment with anti-TNF-α, primarily attributable to the emergence of an immune response targeting the drug itself. This results in decreased concentrations of anti-TNF-α and the detection of anti-anti-TNF-α antibodies in the serum. Immunomodulatory therapies (IMTs) are incorporated to mitigate the immunogenicity against anti-TNF-α medications. While solid evidence-based data regarding their usage is lacking, it is recommended to consider the concurrent use of an immunosuppressant like methotrexate, particularly in cases of uveitis associated with certain pathologies such as Behçet’s disease (BD), juvenile idiopathic arthritis (JIA), and others (51).

## IL inhibitors

Uveitis arises from an imbalance between inflammatory and regulatory mechanisms. Initial acute inflammation is triggered by cells already present in affected tissues, primarily resident macrophages and dendritic cells (52). In autoimmune uveitis, self-reactive T cells exit the thymus and encounter retinal antigens upon reaching the eye. Myeloid dendritic cells exhibit a robust ability to capture antigens, enabling them to activate T cells. Consequently, T lymphocytes differentiate into various subsets—Tregs, Th1, Th17, or Th2—tailored to the encountered antigen and cytokine milieu. Th1 and Th17 cells contribute to inflammatory and autoimmune uveitis, with Th1 cells being pivotal in uveitis development, while Th17 cells assume a significant role in the later/chronic stages. However, induced Treg cells counteract both Th1 and Th17 responses (53–57).

Th1 cells, secreting IL-2, INF-γ, and lymphotoxin, crucially activate macrophages and induce nitric oxide production (52). Th2 cells, secreting IL-4, IL-5, and IL-13, mediate humoral immune responses. A third subset, Th17 cells, discovered and characterized a decade ago, contributes to inflammation by producing pro-inflammatory cytokines such as IL-17, IL-23, and TNFα, recruiting leukocytes from circulation and leading to tissue damage (55, 56).

Experimental uveitis induction is marked by the polarization of early T helper (Th) 0 or Th2-like responses toward Th1 and Th17, while disease resistance is associated with regulatory cells and polarization toward a Th2 pathway. Additionally, the migration of Th1 and Th17 cells to the eye contributes to the breakdown of the blood-retinal barrier, facilitating the recruitment of various leukocytes from circulation (**Figure 1**) (57–59).

### IL-6 inhibitors

Interleukin-6 (IL-6) is a proinflammatory cytokine involved in numerous immune-mediated conditions. It is primarily produced by monocytes, macrophages, B cells, and T cells (60). Elevated intraocular levels of IL-6 are observed in certain conditions like uveitis, central retinal vein occlusion and diabetes (61, 62).

Tocilizumab(TCZ) is a humanized monoclonal antibody that functions by inhibiting IL-6 signaling, thereby preventing IL-6 from binding to its receptor. TCZ has received approval for treating rheumatoid arthritis, Still’s disease, and giant cell arteritis (63–65). STOP trial and APTITUDE study have shown the efficacy of TCZ in non-infectious uveitis. STOP study had included treatment-naive eyes in non-infectious uveitis and APTITUDE study had included eyes refractory to TNF alpha inhibitors in JIA-associated uveitis “ (66, 67). It’s efficacy has also been shown in cases of refractory Bechet’s disease (68). In a retrospective study focusing on macular edema resistant to traditional immunosuppressive medications and biotherapies, TCZ appears notably effective in reducing macular edema. The study found that TCZ facilitated a sustained improvement in macular edema for 80% of the patients (69). In the United States, induction therapy involves a monthly IV regimen of 4 mg/kg, with the option to escalate to 8 mg/kg monthly depending on the therapeutic response. In contrast, in Europe, the approved initial and ongoing dose is 8 mg/kg IV monthly, which may be adjusted to 4 mg/kg if adverse effects arise (70). The majority of adverse events primarily manifest as a heightened susceptibility to infections (8.5%), predominantly affecting the gastrointestinal tract, and elevated liver enzymes (59 to 71%), occasionally leading to acute hepatitis and cytopenia in some instances (71).

The SATURN study investigated sarilumab, an additional anti-IL6 receptor for non-infectious uveitis (NIU). While the study demonstrated its effectiveness in improving visual acuity and reducing macular edema in NIU, adverse effects such as neutropenia and hepatic disorders were reported (72). Other drugs currently undergoing trials include Sarilumab, Sirukumab, Siltuximab, Clazakizumab, Olokizumab, ALX-0061, and MEDI 5117 (70).

### IL-1 inhibitors

Elevated levels of IL-1β have been detected in the aqueous humor of individuals with anterior uveitis (73). Consequently, multiple studies have concentrated on inhibiting IL-1β to treat uveitis. Anakinra, a recombinant monoclonal antibody, targets the IL-1β receptor. Canakinumab, a humanized monoclonal antibody, specifically inhibits IL-1β. The efficacy of anakinra and canakinumab in treating uveitis has been documented, particularly in Behçet’s disease. The median time for therapy response was 6 weeks with anakinra and 3 weeks with canakinumab. Studies have indicated that anti-IL-1 therapy led to improvements in retinal vasculitis lesions and a reduction in uveitis flares. Nevertheless, there was no notable improvement concerning macular thickness and alterations in visual acuity (74). Cantarini et al. proposed that while anakinra effectively managed ocular inflammation, it did not serve as a preventative measure against relapse (75). The majority of adverse events were characterized by bacterial and viral infections, neutropenia and skin reactions at injection sites (76).

### IL-17 inhibitors

Secukinumab is the only anti-IL17 agent explored in uveitis treatment. It was a fully human monoclonal antibody targeting IL-17A. Dick et al. summarized findings from three randomized, controlled trials(SHIELD, INSURE and the ENDURE) in non-infectious uveitis. In these trials, secukinumab was administered subcutaneously to patients previously treated with immunosuppressive drugs. Dosages of secukinumab ranged from 150 mg to 300 mg, given every 2 or 4 weeks. Results across all three studies revealed no significant disparity between treatment and placebo concerning ocular inflammation, relapse rate, or visual acuity enhancement. However, secukinumab led to a notable reduction in the concurrent use of immunosuppressive drugs (77). Nevertheless, the effectiveness of subcutaneous and intravenous secukinumab (at doses of 10 mg/kg every 2 weeks or 30 mg/kg every 4 weeks) has been shown by a few groups in Behçet’s disease (78, 79). Adverse effects such as upper respiratory tract issues (nasopharyngitis) and headaches have been reported. Cases of uveitis reactivation and arthralgia have also been observed (77).

### IL-23 inhibitors

Ustekinumab is a monoclonal antibody targeting the human IL-12/IL-23 p40 subunit, demonstrating efficacy in treating plaque psoriasis, psoriatic arthritis, and inflammatory bowel disease. Interleukin-23 plays a significant role in driving pathology related to Th17 cells (80). Patients with inflammatory bowel disease may receive an initial weight-based intravenous infusion. Several case reports have described the use of ustekinumab in the complete resolution of non-infectious uveitis, including patients with psoriatic arthritis, Crohn’s disease and multiple sclerosis. The medication is administered via subcutaneous injections of 45mg, initially repeated after 4 weeks and subsequently at 8- to 12-week intervals (81, 82). The main adverse effects associated with drugs are gastrointestinal disturbances and infections (83).

## Anti CD-20

Rituximab, a chimeric monoclonal antibody targeting CD20, is approved for the treatment of rheumatoid arthritis. Anti-CD20 rituximab is a monoclonal antibody composed of both murine and human elements, specifically designed to target the CD20 molecule—a tetraspan membrane protein exclusively present on mature B cells’ surfaces. The expression of CD20 undergoes dynamic changes, initially appearing during the immature B cell stage and later diminishing as B cells differentiate into plasma cells. By targeting CD20, Anti-CD20 RTX spares B cell precursor stem cells from destruction. Moreover, most long-lived plasma cells residing in the bone marrow lack the CD20 antigen, thus avoiding their elimination. Consequently, continuous production of immunoglobulins against isoantigens and antibodies against previously encountered pathogens persists. The precise mechanism through which RTX induces B cell death remains incompletely understood, but it is likely a combination of antibody-dependent cell-mediated cytotoxicity, complement-mediated lysis, growth inhibition, and apoptosis. Studies employing mouse models of immunotherapy suggest that antibody-dependent cell-mediated cytotoxicity is the primary mechanism of action. However, these mechanisms operate in a complex manner, with varying responses observed among lymphoma cells at different stages of maturation when exposed to RTX-induced apoptosis and proliferation inhibition (84, 85).

The rationale for targeting B cell depletion in treatment is multifaceted: if autoantibody production plays a pathogenic role, targeting CD20 B cells prevents their differentiation into autoantibody-secreting plasma cells. However, long-lived plasma cells persist in secreting autoantibodies, and memory cells may remain unaffected (84–86).

The efficacy of rituximab has been shown in non-infectious uveitis by substantial evidences. Rituximab has significantly shown improvement in cases of refractory posterior uveitis, Behcet disease and Juvenile idiopathic arthritis cases refractory to TNF-α inhibitors. It has demonstrated significant improvement in the resolution of retinal vasculitis, reducing macular edema, and decreasing the frequency of uveitis flares (87–89). A case report has also shown its efficacy in Vogt Koyangi Harada disease (90). Furthermore, rituximab proved efficacious in managing refractory ANCA-positive vasculitis, showing no notable adverse effects (91). Concerning adverse reactions, there are instances of mild reactions such as hives and flushing, while more severe cases necessitated discontinuation of infusion. Additionally, infection such as pneumonia and herpes zoster have been documented (89).

## Anti CD-25

The interleukin-2 (IL-2) receptor system is a lymphokine receptor system which plays a pivotal role in immune response initiation. Daclizumab (Zenapax) is a humanized monoclonal antibody designed to block the alpha subunit epitope of the IL-2 receptor (CD25), which is situated on activated T cells and various other immune system cells (92). Daclizumab has been utilized safely and effectively at low doses to treat intermediate and posterior uveitis in adults and, to a limited extent, in children (93, 94). Previous research has shown successful outcomes with intravenous (IV) daclizumab as a sparing agent for glucocorticoids and cyclosporine in patients with noninfectious intermediate and posterior uveitis (95). Subsequent studies have confirmed the efficacy of subcutaneous (SC) administration of daclizumab (96). Most recently, it has been demonstrated that high-dose daclizumab effectively controls active intermediate and posterior uveitis (93). Moreover, administering high-dose intravenous daclizumab has shown promising results in decreasing active inflammation in JIA-associated anterior uveitis; nonetheless, patients require vigilant monitoring for potential adverse reactions (97). The commonly reported adverse effects include herpes zoster, rash, and palpitations, as well as liver disorders and elevated transaminases, albeit mostly asymptomatic and self-limiting (97).

## Anti-CD 52

Alemtuzumab, a humanized monoclonal antibody targeting CD52, induces a rapid reduction in circulating T- and B-cell populations upon intravenous administration (98). Its current clinical use extends to refractory chronic lymphocytic leukemia and relapsing multiple sclerosis. Despite its established efficacy in these conditions, there remains limited documentation regarding the role of Alemtuzumab in non-infectious uveitis (NIU), with only a few case reports and series available.

Initially employed in a case of panuveitis resistant to multiple treatments, including cyclosporine A, Alemtuzumab has since demonstrated success in treating refractory Behçet’s disease as well (99, 100). Notably, recent literature includes a report detailing remission achieved with Alemtuzumab treatment in a case of multiple sclerosis featuring bilateral intermediate uveitis and macular edema, unresponsive to alternative therapies (98). Additionally, a case series by Dick et al. has highlighted the favorable outcomes of Alemtuzumab in NIU (101).However, to date, there have been no clinical trials documenting its efficacy in this context.

Reported common side effects of Alemtuzumab encompass localized allergic reactions, mild flu-like symptoms, and gastrointestinal irritation. Nonetheless, there are reports suggesting a potential association between Alemtuzumab use and the development of autoimmune thyroiditis (102, 103).

## mTOR inhibitors

Mammalian target of rapamycin (mTOR) inhibitors have recently garnered interest in ophthalmology and may emerge as viable options for this purpose. These inhibitors constitute a class of immunomodulatory agents that exert their anti-inflammatory effects by impeding T cell function. Among this class are sirolimus (also known as rapamycin) and everolimus (104, 105). These agents work by inhibiting mTOR, a serine/threonine kinase with wide-ranging effects on cellular processes. Specifically, regarding T cells, mTOR inhibitors disrupt signal transduction downstream of the cytokine receptor for IL-2, thereby preventing IL-2 from stimulating T cell proliferation and differentiation. This mechanism may offer therapeutic benefits in the context of uveitis, given that the immune dysfunction in non-infectious uveitis is primarily mediated by T cells (106, 107).

Sirolimus, alternatively referred to as rapamycin, is an immunomodulatory agent derived from bacteria. It suppresses the T cell proliferation by hindering the expression of IL-2, IL-4, and IL-15. This inhibition occurs through the binding of the immunophilin FKBP-12, thus preventing its binding and activation of mTOR. Sirolimus is FDA-approved for renal transplantation (108, 109). Systemic administration of sirolimus is linked to cytotoxic adverse effects, particularly hematological, which may restrict its utility in uveitis treatment (110). Nonetheless, local formulations of the drug for subconjunctival (SCJ) or intravitreal (IVT) injections have been developed and deemed suitable based on preclinical investigations (111). Current clinical trials are directed towards identifying the optimal effective dosage of sirolimus in these formulations (112–114).

The SAVE 2013 trial and its subsequent study, conducted by Ibrahim et al., were Phase I, open-label, randomized clinical trials carried out at a single clinical center in the USA (114). Similarly, SAVE 2 2016 was a Phase II, prospective randomized, open-label, multicentered interventional clinical trial carried out at four clinical centers in the USA (113). Later, SAKURA 2016, the most extensive clinical trial of sirolimus to date, was a Phase III, randomized, double-masked global study conducted across India, European Union Israel, Japan, Latin America, and the USA (112).The details of the study are mentioned in **Table 2**.

## JAK(Janus-associated kinase) inhibitors

JAK inhibitors (Jakinibs) are small molecules designed to impede the Janus kinase family of receptors. JAK-mediated pathways are implicated in the development of various autoimmune conditions such as rheumatoid and psoriatic arthritis, inflammatory bowel disease, and other immune-mediated inflammatory disorders. The JAK family comprises four known members (JAK 1, 2, 3, and TYK2), which belong to the tyrosine kinase family of protein kinases. Tofacitinib and baricitinib represent first-generation JAK inhibitors. Tofacitinib targets JAK 1, JAK 3, and to a lesser extent JAK 2, while baricitinib inhibits JAK 1 and JAK 2 (29).

The effectiveness of tofacitinib, an agent targeting anti-JAK1-JAK3, has been documented in managing refractory ocular inflammation and macular edema (115, 116). Miserocchi et al. published a case series of four cases illustrating the efficacy of JAK inhibitors in JIA. Among these cases, three patients exhibited pan-uveitis, while one had anterior uveitis; all patients also presented with macular edema. Prior to JAK inhibitor treatment, all patients had received anti-TNF-α agents, with three having undergone tocilizumab treatment and three abatacept treatment. One patient received tofacitinib, another received baricitinib (anti-JAK1-JAK2) as monotherapy, and two received baricitinib alongside methotrexate. Efficacy was observed in all patients, manifesting in reduced ocular inflammation and macular edema (29).

Gastrointestinal symptoms, abnormal liver function and rash are common adverse effects observed.

## Abatacept

Abatacept is a recombinant fusion protein comprising a human IgG1 fragment fused to a segment of cytotoxic T-lymphocyte-associated protein (CTLA)-4. This protein binds to CD80 or CD86 on antigen-presenting cells, impeding the costimulatory signal crucial for T-cell activation. In a case series of 7 patients, efficacy of abatacept was observed in JIA patients resistant to TNF-α inhibitors (30). However, a retrospective study of 21 JIA patients showed noted inadequate control of ocular inflammation. Macular edema resolution was observed in only 25% of cases, with no improvement in visual acuity. Corticosteroid tapering led to relapse in all patients treated with abatacept. It is administered either as a weekly 125 mg subcutaneous injection or via intravenous infusions at a dosage of 10 mg/kg (with a maximum of 750 mg) at 0, 2, and 4 weeks, followed by subsequent administrations every 4 weeks (117).

## Interferons

Interferon (IFN)-α and IFN-β, both belonging to the type I IFNs, are naturally occurring cytokines. These IFNs share the same receptor, suggesting their therapeutic effects are quite similar. One observed effect of IFN is the increase in regulatory T cells. IFN-α was the first cytokine to be produced in recombinant form in the early 1980s. Presently, various types of human recombinant IFN-α and IFN-β, administered subcutaneously, are available. Initially, the original formulations required daily injections, but the development of pegylated versions, incorporating polyethylene glycol into the standard structure, allows for significantly lower doses and convenient weekly administration. Pegylation results in a biologically active molecule with enhanced absorption and an extended half-life (118). Main adverse effects reported were gastrointestinal disturbances, infections, myelosuppression, neurological symptoms, flu-like reactions.

### Interferon-α 2a

Recombinant IFN-α-2a finds application in treating chronic hepatitis C and a range of cancers, such as chronic myeloid leukemia and Kaposi sarcoma (119). Dosing schedules vary considerably, contingent upon factors such as the specific indication and whether the formulation is pegylated.

Research conducted on Behçet uveitis refractory to conventional therapy has demonstrated effective inflammation control in over 80% of cases (120–122). In a prospective cohort comprising 50 patients, an overall response rate of 92% was observed, with 82% experiencing no relapses during a three-year follow-up period (32). Deuter et al. and De Simone et al. evaluated the use of IFN-α-2a for refractory macular edema. Administered initially at doses of 3 or 6 million international units subcutaneously per day, with subsequent tapering, the drug achieved response rates of 63% and 100%, respectively (33, 34). Pegylated IFN-α-2a at weekly doses of 90 or 180 μg was retrospectively assessed in 7 patients with persistent uveitic macular edema, resulting in improvement in all cases (123). Recently, IFN-α-2a was employed to treat macular edema secondary to intraocular tuberculosis in 6 patients (124).

### Interferon-α 2b

Both pegylated IFN-α-2a and IFN-α-2b exhibit similar pharmacokinetic profiles (90). A case series involving 4 patients with persistent Behçet uveitis demonstrated the therapeutic efficacy of subcutaneous IFN-α-2b. This treatment regimen involved an initial loading dose of 3 to 4.5 million international units administered daily for 2 weeks, followed by tapering to 3 times weekly (31). Additionally, a retrospective analysis of 35 patients with refractory uveitic macular edema revealed the effectiveness of both IFN-α-2a and IFN-α-2b (125).

### Interferon-β

Recombinant IFN-β is conventionally used for managing multiple sclerosis and is typically administered subcutaneously. Becker et al. scrutinized outcomes in 13 patients afflicted with both multiple sclerosis and uveitis, revealing a decrease in macular edema and enhancements in vision (35).

## Apremilast

Apremilast is a small molecule that specifically targets phosphodiesterase-4. This enzyme is responsible for breaking down adenosine 30, 50 -monophosphate (cAMP), an important intracellular messenger. cAMP plays a role in reducing the synthesis of pro-inflammatory substances like TNF-alpha, IL-23, and IFN-gamma, while promoting the production of anti-inflammatory cytokines such as IL-10 (126). It is an orally administered drug which has been found to be effective for treating oral ulcers in Bechet’s disease (127).

## ACTH analogues

ACTH belongs to a family of molecules known as melanocortins (MC), including ACTH, alpha-, beta-, and gamma-melanocyte stimulating hormone (MSH), which are naturally produced by the cleavage of a larger precursor called pro-opiomelanocortin (128, 129). This cleavage primarily occurs in the pituitary gland as part of the HPA axis. Recent studies have shown that immune cells at sites of inflammation can also produce MCs (130). One of ACTH’s prominent anti-inflammatory mechanisms involves stimulating the adrenal glands to produce glucocorticoids (131).

Five melanocortin receptors (MCRs) have been identified and are found in various cell types, including immune cells such as macrophages, mast cells, neutrophils, and lymphocytes. These receptors exhibit varying affinities for different melanocortins, but ACTH has demonstrated high affinity for all five receptors (132). Among these receptors, MCR2 primarily mediates the induction of cortisol production by ACTH, while MCR 1, 3, and 5 are involved in regulating ACTH’s immunomodulatory effects independently of the HPA axis (133) (128). These effects include reducing cytokine synthesis, inhibiting leukocyte transmigration, and generating local anti-inflammatory signals at sites of inflammation. At the molecular level, the inhibition of nuclear factor-kappa B (NF-kappa B) is a key mechanism underlying the broad anti-inflammatory effects of melanocortin molecules. NF-kappa B regulates the expression of various pro-inflammatory cytokines, receptors, adhesion molecules, and chemokines (134, 135). Agarwal et al. documented the usage case of ACTH gel in young male with uveitis (136). A phase 2 trial showing the efficacy of ACTH gel in non-infectious uveitis is underway. (NCT02931175).

## Immunoglobulins

Intravenous immunoglobulin (IVIG) is a naturally derived polyclonal human IgG obtained from plasma donors. This preparation exerts its affects through various mechanisms, including the modulation of cytokine synthesis and secretion, autoantibodies suppression, inhibition of complement activation and interactions with major histocompatibility complex class 1 molecules and adhesion molecules (137). IVIG finds utility in treating immunodeficiencies and systemic inflammatory conditions. Favorable outcomes have been noted with IVIG administration in Behcet uveitis, VKH syndrome, birdshot retinochoroidopathy, and refractory non-infectious uveitis (NIU) (138–141). The dosing of IVIG varies, but typically involves cycles of 1.5 to 2.5g/kg over three days, repeated at intervals ranging from 2 weeks to 2 months or longer (141, 142). The adverse effects noted with IVIG are systemic HTN, headache, rashes, fever, myalgia, and thrombosis (138, 139, 141–143).

## Non-viral ocular gene therapy (pEYS606)

pEYS606, a plasmid DNA devoid of antibiotic selection genes, encodes a fusion protein that links the extracellular domain of the soluble p55 TNF-α receptor to the human IgG1 Fc domain (known as hTNFR-Is/hIgG1 or Protein 6), exhibiting high affinity for human TNF-α. This plasmid is designed for non-viral gene transfer into the ciliary muscle of the eye. It functions to neutralize the activity of TNF-α and is introduced into the eye using the proprietary Eyevensys Electrotransfection System. The therapy is still undergoing two trials. EYS606-CT1 Trial (EU) A phase 1/2, open-label, multicenter, dose-escalation study was conducted to assess the safety and tolerability of EYS606 in patients with chronic non-infectious uveitis. An ELECTRO trial which is A 48 48-week study to Evaluate the Efficacy and Safety of Two EYS606 Treatment Regimens in Subjects With Active Chronic Non-infectious Uveitis (144–146).

## Intravitreal therapy

Corticosteroids, immunomodulating agents, and intravitreal TNF-α agents have been employed in the treatment of non-infectious uveitis. Various corticosteroids used in this context include triamcinolone acetonide (IVTA), dexamethasone, and fluocinolone acetonide implants. IVTA is commonly utilized for managing uveitic macular edema in non-infectious uveitis. Ozurdex (0.7 mg), a dexamethasone implant, is approved for treating macular edema secondary to non-infectious uveitis and has demonstrated efficacy in reducing significant vitreous haze.

Fluocinolone acetonide implants are available in different doses: 0.59 mg (Retisert^®^, Bausch and Lomb, Inc.), 0.18 mg (Yutiq^®^, Eyepoint Pharmaceuticals, Inc.), and 0.19 mg (Iluvien^®^, Alimera Sciences, Inc.). Despite the short systemic half-life of fluocinolone acetonide (FA), non-biodegradable FA implants release the steroid at a stable rate for up to 3 years, offering a much longer duration of action compared to dexamethasone and triamcinolone acetonide. POINT trial showed that one of the primary drawbacks of intravitreal corticosteroid therapy is the rise in intraocular pressure, necessitating the prescription of anti-glaucoma medications to manage it (147).

The efficacy of a single intravitreal injection of 400 μg methotrexate (MTX) in 0.1 ml has been investigated in a case series involving 15 patients with unilateral exacerbations of non-infectious posterior uveitis and/or macular edema. Although intravitreal 400 μg MTX appears to be a safe and effective alternative to intravitreal corticosteroids, larger randomized clinical trials with longer follow-up periods are required to establish its therapeutic role in managing non-infectious uveitis. Compared to intravitreal corticosteroids, intravitreal MTX is less likely to be associated with intraocular pressure elevation and cataract development, though corneal epitheliopathy is a reported side effect.

One of the main disadvantages of intravitreal MTX is its short duration of action, necessitating repeated injections, unlike sustained-release corticosteroid implants. Intravitreal sirolimus has also been under trial in various doses, while intravitreal infliximab and adalimumab have been explored, though studies provide contrasting results on their efficacy as therapeutic alternatives for refractory non-infectious posterior uveitis (148).

## Conclusion

Over the past two decades, biologic agents have brought about a transformative shift in the immunological treatment of non-infectious uveitis (NIU). The most recent guidelines for managing uveitis advocate for a stepwise approach, commencing with the use of topical, periocular, and systemic corticosteroids (CS), progressing to immunomodulatory therapy (IMT), and ultimately considering the adoption of biologic therapy, preferably a TNF-α inhibitor. Despite the widely recognized side effects associated with CS, they remain fundamental in the treatment of acute episodes and exacerbations. Moreover, the adoption of new therapeutic options is currently hindered by their elevated cost and the need for further investigation into various aspects, such as follow-up protocols and monitoring procedures. However, it would not be surprising if, with the accumulation of new data, biologic agents are eventually recommended as the primary treatment modality for certain forms of uveitis. Additionally, ongoing research has unveiled novel insights, presenting new therapeutic avenues targeting immune components as potential innovative strategies for managing NIU.

In experimental models, innovative approaches such as exosome therapy, nanobodies, and IL-27 exhibit promising potential for treating non-infectious uveitis by regulating immune responses and diminishing inflammation. These therapies work by enhancing regulatory mechanisms and restraining overactive immune reactions, thereby preserving immune tolerance and managing ocular inflammation. Collectively, these advanced therapies present promising strategies for improving the management of non-infectious uveitis, offering potential for more effective and targeted treatments.

## Author contributions

KC: Conceptualization, Data curation, Formal analysis, Investigation, Methodology, Project administration, Resources, Software, Visualization, Writing – original draft, Writing – review & editing. MT: Conceptualization, Formal analysis, Investigation, Methodology, Project administration, Resources, Software, Supervision, Validation, Visualization, Writing – original draft, Writing – review & editing.
